# A mixed methodology, non-interventional study to evaluate the use of direct oral anticoagulants in UK clinical practice for patients with a first stroke associated with non-valvular atrial fibrillation: study protocol

**DOI:** 10.1186/s12883-019-1530-0

**Published:** 2019-11-29

**Authors:** Yaqoob Bhat, Anand Dixit, Amit Mistri, Bhavini Patel, Sadat Haider Quoraishi, James Uprichard

**Affiliations:** 1 Aneurin Bevan University Health Board, St Cadoc’s Hospital, Lodge Road, Caerleon, Newport, NP18 3XQ UK; 2 Newcastle upon Tyne Hospitals NHS Foundation Trust, Freeman Hospital, High Heaton, Newcastle Upon Tyne, NE7 7DN UK; 3 University Hospitals of Leicester NHS Trust, Infirmary Square, Leicester, LE1 5WW UK; 4 St George’s University Hospitals NHS Foundation Trust, Blackshaw Road, Tooting, London, SW17 0QT UK; 5 Medical Department, Daiichi Sankyo UK Ltd, Building 1, Chalfont Park, Gerrards Cross, SL9 0GA UK

**Keywords:** Non-valvular atrial fibrillation, Ischaemic stroke, CHA_2_DS_2_-VASc risk score, Anticoagulant, Direct oral anticoagulants, Treatment adherence, Treatment satisfaction, Hospital resource use

## Abstract

**Background:**

Treatment with anticoagulants, including direct oral anticoagulants (DOACs), should be considered for patients diagnosed with atrial fibrillation (AF) deemed at risk of ischaemic stroke. There are limited real world data related to the characteristics of patients with non-valvular AF who were not taking anticoagulants at the time of first ischaemic stroke and their subsequent DOAC treatment for the secondary prevention of stroke. Furthermore, little is known about patient adherence and experiences of DOAC treatment, especially for patients with non-valvular AF receiving DOAC therapy for the secondary prevention of stroke.

**Methods:**

This is a UK mixed methodology, non-interventional study, involving retrospective and prospective medical record reviews and a prospective patient survey, in progress in six UK National Health Service secondary/tertiary care centres. The study comprises two groups of patients. Group 1 will include 300 eligible consenting patients with a first ischaemic stroke associated with non-valvular AF untreated with anticoagulants in the 12 months prior to stroke. Group 2 will include a subgroup of 150 patients from Group 1 initiated on one of the DOACs targeting activated Factor X (*n* = 50 on apixaban, n = 50 on edoxaban and n = 50 on rivaroxaban). The primary endpoint of the study is the CHA_2_DS_2_-VASc Risk Score prior to initiation of anticoagulation for patients included in Group 1. Secondary endpoints to be evaluated in Group 1 include patient demographics, clinical characteristics, relevant medical history, anticoagulant therapy initiated for secondary prevention of stroke, and relevant concomitant medication. Secondary endpoints to be evaluated in Group 2 include the time between stroke and DOAC initiation; prescribing of DOACs, other anticoagulants and concomitant medication; clinical assessments and hospital resource use; patient reported outcome measures, including the Morisky Medication Adherence Scale questionnaire and the Treatment Satisfaction Questionnaire for Medication.

**Discussion:**

This mixed methodology study will provide new real world insights into the characteristics and management pathways and patient-reported experiences of this important group of patients. It is anticipated that the results of this study will provide the medical community and patients with important information to inform clinical decision-making and help facilitate meaningful improvements in the care of patients with non-valvular AF.

## Background

Atrial fibrillation (AF) is the most common chronic cardiac arrhythmia, which often remains undiagnosed and is associated with a significantly increased risk for thromboembolic stroke [1–3]. It has been estimated by the Stroke Association that approximately 20% of the more than 100,000 strokes occurring annually in the United Kingdom (UK) are associated with AF [4]. The UK National Institute for Health and Care Excellence recommends that anticoagulation is considered for patients diagnosed with AF who are deemed to be at risk of stroke, as assessed by the CHA_2_DS_2_-VASc score [5, 6].

Direct oral anticoagulants (DOACs) are a class of oral anticoagulants that have been developed and introduced to healthcare systems across the world over the past decade. The first DOAC to market, dabigatran, is a direct thrombin inhibitor which, like warfarin, has been the subject of numerous real world observational research studies [7, 8]. The three DOACs most widely used currently in the UK for the primary and secondary prevention of stroke in patients with non-valvular AF (the most common form of AF) are direct inhibitors of activated factor X (anti-FXa DOACs, namely apixaban, edoxaban and rivaroxaban) [5, 9–11]. Although some real world studies have evaluated the use and effectiveness of apixaban and rivaroxaban in patients with AF [12–14], there are limited data related to the characteristics of patients who were not taking anticoagulants at the time of first ischaemic stroke. There are also limited data related to use of anti-FXa DOACs in routine clinical practice since the introduction of edoxaban in 2015, the last DOAC to market in the UK. Furthermore, while guidance on prescribing each of the anti-FXa DOACs is available [9–11], this may not reflect how they are prescribed and taken by patients treated in routine clinical practice. Patient-related factors, including adherence, how patients perceive the benefits of treatment (including their experience and satisfaction with the treatment and overall care) may impact on treatment effectiveness. However, little is known about the extent to which patients adhere to anti-FXa DOAC treatment and their perceptions of anti-FXa DOAC treatment, especially for patients with AF receiving DOAC therapy for the secondary prevention of stroke.

### Methods/design

#### Aim and objectives

The aim of this mixed-methodology study is to evaluate the real world use of anticoagulation, in particular anti-FXa DOAC treatment (apixaban, edoxaban and rivaroxaban) in routine UK clinical practice in terms of patient characteristics, management pathways and patient-reported experiences of anticoagulation initiated following a first ischaemic stroke associated with non-valvular AF for patients who were not receiving anticoagulant therapy in the 12 months prior to the stroke.

The primary objective of this study is to describe the demographics, clinical characteristics and medical history of patients with AF presenting with a first ischaemic stroke who were not treated with anticoagulants in the 12 months prior to the stroke, according to the type of anticoagulant treatment subsequently prescribed for secondary stroke prevention (apixaban, edoxaban, rivaroxaban, warfarin, dabigatran, other anticoagulants or a documented decision not to initiate anticoagulation). Secondary study objectives will be evaluated only in patients treated with anti-FXa DOACs (apixaban, edoxaban, rivaroxaban), including describing the management pathways of patients initiated on anti-FXa DOAC treatment for secondary prevention of stroke; describing hospital resource use and the clinical assessments associated with anti-FXa DOAC treatment; describing patient-reported adherence to anti-FXa DOAC treatment; and describing patient-reported experience and satisfaction with anti-FXa DOAC treatment.

### Study design and setting

**Fig. 1:**
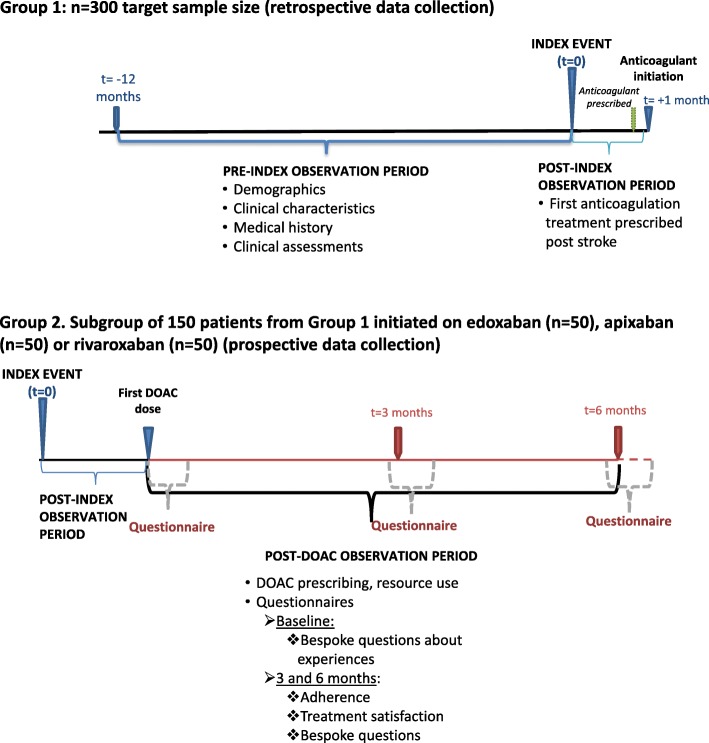
Study design and observation periods. **Group 1**: retrospective data collection for 300 eligible and consenting patients who present with first stroke associated with non-valvular atrial fibrillation (index event). The pre-index observation period is the 12-month period prior to the index event. The post-index observation period is the one month period following the index event **Group 2:** Prospective data collection for the first 150 eligible and consenting patients from Group 1 who commence treatment with apixaban (*n* = 50), edoxaban (*n* = 50), or rivaroxaban (*n* = 50). The post-DOAC observation period is from initiation of anti-FXa DOAC treatment up to a maximum of 7 months (6 months + 1 month window for completion of questionnaires) post-initiation. The baseline questionnaire will be completed within one month following anti-FXa DOAC initiation. The three-month questionnaire will be completed within the period beginning one week prior to and ending one month after the three-month post-initiation time point. The six-month questionnaire will be completed within the period beginning one week prior to and ending one month after the six-month post-initiation time point

This is a UK mixed methodology, non-interventional study, involving retrospective and prospective medical record reviews and a prospective patient survey, which is in progress in six UK National Health Service (NHS) secondary and tertiary care centres. Study centres have been selected to provide good geographic representation of centres experienced in prescribing apixaban, edoxaban and rivaroxaban for the secondary prevention of ischaemic stroke in patients with non-valvular AF. The key elements of study design are summarised in Fig. 1. The index event is the diagnosis of first ischaemic stroke associated with AF. The pre-index observation period is the 12-month period prior to the index event. The post-index observation period is the one month period following the index event. The post-DOAC observation period is the period from initiation of anti-FXa DOAC treatment up to a maximum of 7 months (6 months + 1 month window for completion of questionnaires) post-initiation.

### Patients

Two groups of patients will be included in the study. Group 1 will comprise 300 patients with a first ischaemic stroke that was associated with non-valvular AF who were not treated with anticoagulants in the 12 months prior to the stroke. The primary study objective will be evaluated in Group 1. Group 2 will be a subgroup of 150 patients from Group 1, comprising 50 patients initiated on apixaban, 50 patients initiated on edoxaban and 50 patients initiated on rivaroxaban. The secondary study objectives will be evaluated in Group 2.

Patients will be eligible for inclusion in Group 1 if they are aged ≥18 years at time of presentation to the study centre with a first ischaemic stroke which, in the clinician’s opinion, is associated with non-valvular AF. Eligible patients must not have been prescribed anticoagulants for any indication in the 12 months prior to stroke diagnosis, or have a history of haemorrhagic stroke or transient ischemic attack or have a severe cognitive or emotive deficit at the time of consent. Patients will be ineligible for inclusion in Group 1 if their medical records are not available for review or if they are unwilling or unable to give written informed consent for researcher access to their medical records. Patients in Group 1 will be eligible for inclusion in Group 2 if they are initiated on apixaban, edoxaban or rivaroxaban for secondary prevention of stroke. Patients in Group 1 will be ineligible for inclusion in Group 2 if they are unwilling or unable to complete the patient-reported questionnaires.

Patients will be identified and recruited prospectively during a one-year period from the point of approval to commence recruitment at participating centres. Potentially eligible patients will be identified by members of the direct care team from local department databases. Patients fulfilling the Group 1 eligibility criteria will be approached to provide written informed consent for retrospective data collection from their medical records by members of the direct care team. Patients will be approached in consecutive chronological order, according to the date of stroke diagnosis, until the required sample size or the centre-specific recruitment target is reached. The first 50 eligible and consenting patients initiated on either apixaban, edoxaban or rivaroxaban at any of the study centres during the one year recruitment period will also be included in Group 2. Although centres included in the study will have experience of prescribing all three DOACs of interest, it is likely that some centres will favour a particular anti-FXa DOAC (although feasibility exercises suggest that the favoured DOAC will vary between centres). Recruitment of patients into Group 2 will be evaluated on an ongoing basis to monitor whether a study centre is predominately favouring one type of DOAC. To ensure there is balanced enrolment of patients into the Group 2 apixaban, edoxaban and rivaroxaban sub-groups, centre enrolment targets may be implemented to ensure balanced representation across centres in order to minimise bias.

### Data sources and data collection

#### Retrospective medical record review

Data will be collected retrospectively from the hospital medical records of patients included in Group 1 in order to describe patient demographics and clinical characteristics and relevant medical history, including the components required to assess the CHA_2_DS_2_-VASc risk score (age, sex, history of congestive heart failure, hypertension, diabetes mellitus, stroke/transient ischaemic attack/thromboembolism, vascular disease). Where relevant, primary care data will also be requested from patients’ General Practitioners (GP). Data on the type of anticoagulant treatment prescribed for secondary prevention of stroke will be collected retrospectively for the one month post-index observation period. These time periods were chosen to ensure sufficient time to capture relevant data related to patient demographic and clinical characteristics prior to first stroke, accounting for variations in appointment timing and scheduling seen in routine clinical practice. A prior scoping exercise involving five healthcare professionals suggested that the data required to evaluate the primary endpoint (summary of CHA_2_DS_2_-VASc risk characteristics) are likely to be routinely recorded. Furthermore, the CHA_2_DS_2_-VASc risk score has been shown to be a useful predictor of patient outcomes over a 12 month period [15] and so a 12 month pre-index observation period was deemed appropriate. All data obtained from medical records will be recorded in electronic case report forms (eCRFs) designed specifically for the study, using a secure web-based electronic data capture system.

### Prospective medical record review

Details of anti-FXa DOAC treatment and hospital resource utilisation will be sourced prospectively from the hospital medical records of patients included in Group 2 for the six-month period following initiation of anti-FXa DOAC treatment and entered into the eCRFs. In certain cases, it may be necessary to contact the patient’s GP to obtain information from primary care records. However, it is anticipated from feasibility assessments that important information about the patient’s treatment will be captured within the secondary care records, as it is likely that patients will be referred back into secondary care should any adjustment to their anticoagulant treatment be needed.

### Prospective patient survey

Patient-reported outcome data will be collected prospectively from patients in Group 2 via questionnaires administered at baseline (initiation of anti-FXa DOAC treatment + 1 month), and at 3 months (− 1 week/+ 1 month) and 6 months (− 1 week/+ 1 month) after initiation of anti-FXa DOAC treatment for secondary prevention of stroke. These time-points were chosen in order to allow an adequate amount of time between assessment points for DOAC treatment to take effect. Questionnaires will be administered face-to-face if patients are attending clinic at the relevant time point; they will be reminded of the study requirements and will be given the opportunity to decide whether or not they would still like to take part, prior to the questionnaire being administered. Otherwise questionnaires will be administered via telephone, which is a methodology for administration of patient questionnaires that has been used in prior studies, including a study relating to edoxaban treatment [16]. Where questionnaires are administered by telephone, the questionnaires will be posted to patients by members of their direct care team at the relevant time points to give patients an opportunity to contemplate their responses and complete the questionnaires in advance. Patients will be contacted by telephone to advise them that the questionnaire has been posted, to remind them of the study requirements and to ensure that they are still willing to participate in the study. A member of the direct care team or an external researcher will contact patients at an agreed date and time to collect their responses via telephone. The questions will be read to patients as written on the paper version and patients’ responses will be entered directly into the eCRF.

### Study endpoints and variables

The primary endpoint of the study is the CHA_2_DS_2_-VASc Risk Score (scores range from 0 to 9) prior to initiation of anticoagulation for patients included in Group 1. Secondary endpoints to be evaluated in patients in Group 1 include patient demographics (including age, sex, body mass index [BMI], smoking status, alcohol use, ethnicity) and clinical characteristics (including time from AF diagnosis until stroke, recorded stroke severity, location of presentation with stroke [e.g. Emergency Department (ED), inpatient, outpatient, GP], and key clinical and laboratory evaluations at the time of stroke (including renal function, liver function and blood parameters), relevant medical history (including history of congestive heart failure, hypertension, diabetes mellitus, vascular disease [including myocardial infarction, aortic plaque, peripheral vascular disease], renal disease, hepatic disease), anticoagulant therapy initiated for secondary prevention of stroke, and relevant concomitant medication at the time of stroke and prescribed in the one month period after stroke. Secondary endpoints to be evaluated in patients in Group 2 include: the time between index event and date of anti-FXa DOAC initiation; anti-FXa DOACs and other anticoagulants prescribed during the post-DOAC observation period (including dose, and dosing frequency and reason for choice of first DOAC treatment, changes in dose and reasons for dose changes, DOAC discontinuation, time from initiation to discontinuation of first DOAC and reason(s) for discontinuation, anticoagulant treatments subsequently prescribed and reason(s) for choice/discontinuation); relevant medications newly prescribed in the post-DOAC observation period; clinical assessments (types and results of clinical assessments); hospital resource use (number, type [outpatient, inpatient, ED, other; planned or unplanned], setting [face-to-face, telephone, other], reason for hospital visits/consultations, length of stay for inpatient admissions, time to first follow-up visit from date of DOAC initiation) during the post-DOAC observation period.

Patient reported outcome measures include the Morisky Medication Adherence Scale (MMAS-8) questionnaire [17] and the Treatment Satisfaction Questionnaire for Medication (TSQM) questionnaire [18]. The MMAS-8 is a short, eight-item measure of treatment adherence that has previously been used to assess adherence to DOAC treatment in patients with venous thromboembolism [19]. The MMAS has been used widely in different diseases, populations and countries and has been administered face-to-face or via telephone [20–22]. The MMAS-8 is quicker to complete and has a higher degree of concordance with more objective measures of treatment (such as pharmacy fill data or electronic monitoring devices) than many other self-report measures [20]. In order to gain additional insight into recent levels of treatment adherence, patients will also be asked to report whether, how and how often they had taken their current anti-FXa DOAC treatment in the past seven days using a bespoke questionnaire. The TSQM is a widely validated general measure of treatment satisfaction which has been used for a variety of treatments and disease areas, including for measuring levels of patient satisfaction with anticoagulation treatment [23], and has been administered face-to face or via telephone [22]. The bespoke questionnaire will be administered to gain an understanding of the general experiences of patients with regard to other aspects of their care, such as communication, education about anti-FXa DOAC treatment and overall hospital experience.

### Ethical considerations

Patients will be identified in all study documents and questionnaires by a unique study identification number that will be used to link multiple study records for each participant. No personally identifiable information on any participant will be collected or removed from the participating centres in order to preserve patient confidentiality. Patients will provide written informed consent according to the Research Ethics Committee (REC)-approved protocol and consent materials. Only patients providing written informed consent will be included in the study and no data collection on an individual will take place until their written informed consent has been obtained. Participants will have the right to withdraw their consent at any time, without giving a reason and without prejudice to their normal care. NHS management approval for local conduct of the study and the sharing of pseudonymised patient data will be sought via the Research and Development department in each participating centre.

### Statistical methods

#### Study sample size

Study endpoints will be described for the overall patient population and in patients stratified by anticoagulant treatment received; however, no formal comparisons between groups will be made. Therefore the required sample size was estimated based on precision (95% confidence limits) rather than statistical power. For Group 1 a sample size of 300 patients is considered sufficient to reliably describe the demographics and clinical characteristics of the study population. Based on feasibility interviews with a small sample of UK clinicians it is anticipated that approximately 80% of patients in Group 1 are likely to be prescribed a DOAC for secondary prevention of stroke. Therefore, a sample size of 150 patients in Group 2 distributed evenly between patients initiated on apixaban, edoxaban and rivaroxaban is considered feasible. A sample size of 50 patients treated with each of the anti-FXa DOACs is considered sufficient to give reliable estimates of patient adherence based on scores from a previously published study of DOAC adherence [24]. For a sample of 50 patients with a mean adherence score of 7.2 and a standard deviation of 1.2, the lower and upper confidence limits are expected to be between 6.9 and 7.5, indicating the sample size is sufficient to provide a reliable estimate of mean adherence score.

### Data analysis

Statistical analyses, including any data preparation prior to analysis, will be carried out using Stata v14 (StataCorp LLC) and Microsoft Excel (2010). Data from all centres will be pooled for analysis. Study endpoints will be presented as descriptive statistics of central tendency (median; arithmetic mean or geometric mean) and dispersion (interquartile range [IQR] and/or range; standard deviation [SD] and/or 95% confidence intervals [95% CI]) and/or frequencies and percentages, as appropriate for the type and distribution of the variables.

Total scores for the validated patient-reported questionnaires will be derived in accordance with published scoring algorithms for each measure [17, 18, 25]_._ Analyses will be conducted using only the results of those patients with data available.

### Subgroup and sensitivity analyses

All endpoints evaluated in Group 2 will be stratified according to the anti-FXa DOAC first prescribed (apixaban, edoxaban, rivaroxaban). Patient-reported outcomes will also be analysed separately for patients remaining on first anti-FXa DOAC treatment and those switching DOAC treatment during the post-DOAC observation period, in order to explore the relationship between patient-related factors and decisions to switch treatments. No sensitivity analyses are planned.

### Limitations of the research methods

Based on feasibility assessments it is expected that all relevant data to evaluate the CHA_2_DS_2_-VASc risk score will be available for all patients. However, it is possible that some or all of the required components needed to calculate the CHA_2_DS_2_-VASc score will be unavailable for some patients, which may introduce bias in terms of a reduction in the sample size and may lead to underestimation of stroke risk. The exclusion of patients with persisting cognitive or emotive deficit at the time of consent may lead to an underestimation of hospital resource use; patient-reported outcomes may also not be representative of the wider patient population. In addition, primary care data following discharge home from hospital after stroke and/or data relating to patient management prior to stroke may also not be available for all patients, which may affect the related endpoints. This is a descriptive study and no comparative analyses or analyses to control for confounding factors will be carried out. As such, no inferences about causal relationships between different DOACs and study endpoints and how treatments compare to each other will be made. For patient-reported measures, the data will rely on the completeness of the answers provided by participants, which will not be queried or otherwise followed up to clarify inconsistencies or omissions. The importance of providing honest and complete responses at each time point will be emphasised to patients; data may however be subject to patient recall and reporting bias. Patient-reported data based on recall (for example, for adherence to medication) may also be subject to recall bias and/or reporting bias.

## Discussion

Despite the increased risk of stroke in patients with AF, recent data from the Sentinel Stroke National Audit Programme indicate that more than 40% of patients with known AF were not taking anticoagulants at the time of presentation with stroke in the period from April 2017 to March 2018 [26]. This mixed methodology study will provide new real world insights into the characteristics, management pathways and patient-reported experiences of patients who were not receiving anticoagulant therapy in the 12 months prior to first ischaemic stroke associated with non-valvular AF. The mixed methodology approach, involving a combination of retrospective and prospective data collection, will enable collection of data from medical records before and after first stroke in a timely fashion and also enable patient-reported data to be collected at relevant time-points during DOAC treatment. It is anticipated that the results of this study will provide the medical community and patients with important information to inform clinical decision-making and help facilitate meaningful improvements in the care of patients with non-valvular AF.

## Abbreviations

AF: atrial fibrillation
BMI: body mass index
CI: confidence interval
DOAC: direct oral anticoagulant
eCRF: electronic case report form
ED: Emergency Department
GP: General Practitioner
FXa: activated factor X
IQR: interquartile range
MMAS: Morisky Medication Adherence Scale
NHS: National Health Service
REC: Research Ethics Committee
SD: standard deviation
TSQM: Treatment Satisfaction Questionnaire for Medication
UK: United Kingdom
